# Synergism of Heat Shock Protein 90 and Histone Deacetylase Inhibitors in Synovial Sarcoma

**DOI:** 10.1155/2009/794901

**Published:** 2009-03-24

**Authors:** Anne Nguyen, Le Su, Belinda Campbell, Neal M. Poulin, Torsten O. Nielsen

**Affiliations:** ^1^Jack Bell Research Centre, Vancouver Coastal Health Research Institute, The University of British Columbia, 553 - 2660 Oak Street, Vancouver, BC, Canada V6H3Z6; ^2^Department of Radiation Oncology, BC Cancer Agency, 600 West 10th Avenue, Vancouver, BC, Canada V5Z4E6

## Abstract

Current systemic therapies have little curative benefit for synovial sarcoma. Histone deacetylase (HDAC) inhibitors and the heat shock protein 90 (Hsp90) inhibitor 17-AAG have recently been shown to inhibit synovial sarcoma in preclinical models. We tested combinations of 17-AAG with the HDAC inhibitor MS-275 for synergism by proliferation and apoptosis assays. The combination was found to be synergistic at multiple time points in two synovial sarcoma cell lines. Previous studies have shown that HDAC inhibitors not only induce cell death but also activate the survival pathway NF-*κ*B, potentially limiting therapeutic benefit. As 17-AAG inhibits activators of NF-*κ*B, we tested if 17-AAG synergizes with MS-275 through abrogating NF-*κ*B activation. In our assays, adding 17-AAG blocks NF-*κ*B activation by MS-275 and siRNA directed against histone deacetylase 3 (HDAC3) recapitulates the effects of MS-275. Additionally, we find that the NF-*κ*B inhibitor BAY 11-7085 synergizes with MS-275. We conclude that agents inhibiting NF-*κ*B synergize with HDAC inhibitors against synovial sarcoma.

## 1. Introduction

Synovial sarcoma is an aggressive malignancy, typically occurring in the soft tissues of young adults, comprising up to 10% of adult sarcomas in some series [1]. Current therapies for synovial sarcoma involve surgical resection followed by either radiotherapy and/or chemotherapy (such as doxorubicin) [2], with 5-year metastasis-free survival rates between 48% and 68% [3–5]. The chromosomal translocation t(X;18)(p11.2;q11.2), resulting in an SYT-SSX fusion oncoprotein, is demonstrable in almost all cases [1], but its molecular function is unclear, and it is not directly targeted by established drugs.

The heat shock protein 90 (Hsp90) inhibitor 17-(allylamino)-17-demethoxygeldanamycin (17-AAG) is effective against synovial sarcoma in vitro [6]. Hsp90 is a chaperone assisting in the folding of client proteins such as CDK-4, Raf-1 [7], HER2 [8], mutant p53 [9], Bcr-Abl [10], IKK [11], and RIP [12] which play important roles in oncogenic cell growth, division, and survival. Hsp90 inhibition results in degradation of its client proteins, inhibition of tumor growth, induction of differentiation and activation of apoptosis [13]. The Hsp90 inhibitor 17-AAG has completed phase I clinical trials [14] and is in phase II for several malignancies including metastatic melanoma, breast cancer, and ovarian cancer [15].

The HDAC inhibitor romidepsin (FK228; depsipeptide) has also been demonstrated to be effective against synovial sarcoma models, where nanomolar levels cause histone acetylation, inhibition of growth, and invasion in cell cultures and mouse xenografts [16]. We have confirmed these findings using the HDAC inhibitors trichostatin A, romidepsin, and MS-275 on synovial sarcoma cell lines [17, 18]. HDAC activity acts within transcription factor complexes to suppress transcription at target loci, including tumor suppressors and genes driving differentiation [19, 20] by decreasing net acetylation of histones. HDAC inhibitors lead to acetylation of several other proteins, including p53 [21], NF-*κ*B [22], and Hsp90 [23] which have roles in cellular growth, survival, and protein folding. Several HDAC inhibitors (including MS-275; an orally bioavailable agent) are currently involved in phase I and II clinical trials of malignancies including leukemias, lymphomas, melanomas, and refractory solid tumors [24]. In synovial sarcoma, Ito et al. have shown that the SYT partner of the SYT-SSX fusion oncoprotein interacts with a HDAC complex, providing a mechanism for specific activity of HDAC inhibitors in this disease [25]. Furthermore, we have recently shown that HDAC inhibitor action reverses polycomb-mediated epigenetic suppression of SYT-SSX target genes [26].

The evidence that both 17-AAG and HDAC inhibitors are individually effective against synovial sarcoma raises the question of whether combinations would be synergistic. To date, synergy studies using Hsp90 inhibitors and various HDAC inhibitors (SAHA, sodium butyrate, and cinnamic hydroxamic acid analog) have shown positive results in variety of human leukemia cells [10, 27, 28]. However, in other models, the combination appears antagonistic; Huang et al. reported that pretreatment with the Hsp90 inhibitor geldanamycin before addition of trichostatin A averted death in COS-7 cells [29], and Yang et al. found that coadministration of romidepsin with 17-AAG had neither synergistic nor additive effects on RUNX1-ETO levels in Kasumi-1 leukemic cells [30]. As each type of agents is individually effective against synovial sarcoma cells, and synergy could potentially be of great benefit to patients, in this study, we seek to test combinations of 17-AAG with the HDAC inhibitor MS-275 for efficacy against synovial sarcoma.

A possible mechanism for synergy between HDAC and Hsp90 inhibitors involves effects on the survival pathway NF-*κ*B. NF-*κ*B is a transcription factor constitutively activated in many cancer models [31], wherein it confers resistance to apoptosis and promotes cell survival [32], angiogenesis, and invasion [33]. Expression profiling studies by us and others have shown that RIPK4, an activator of NF-*κ*B [34], is highly expressed within synovial sarcoma primary tumor samples [35]. HDAC3 regulates acetylation of the NF-*κ*B subunit RelA, thereby reducing its transcriptional activity [22]. Other studies have confirmed that HDAC inhibitors induce NF-*κ*B activity; an effect which diminishes the lethality of these drugs against lung cancer cell lines [36]. In contrast, 17-AAG has been shown to be an effective inhibitor of the NF-*κ*B pathway [12]. In this work, we also investigate if 17-AAG can synergize with HDAC inhibitors by reducing the activation of NF-*κ*B.

## 2. Materials and Methods

### 2.1. Reagents

17-AAG was provided under the terms of a materials transfer agreement (MTA) with the Developmental Therapeutics Branch of the National Cancer Institute (Bethesda, Md, USA) through Kosan Biosciences (Hayward, Calif, USA). MS-275 was provided under the terms of an MTA by Schering AG (Berlin, Germany) through Berlex Pharmaceuticals (Montville, NJ, USA). RPMI 1640, fetal bovine serum, and trypsin were purchased from Life Technologies (Invitrogen, Mississauga, ON, Canada). siRNA targeting HDAC3 was obtained from Santa Cruz Biotechnology (Santa Cruz, Calif, USA), catalog number sc-35538; a 50 ng dose was confirmed by western blot to knock down HDAC3 in synovial sarcoma cells by 71% at 24 hours relative to scrambled siRNA control. BAY 11-7085 was purchased from Calbiochem (San Diego, Calif, USA). The pNF-*κ*B-Luc plasmid, containing the firefly luciferase (luc) gene from *photinus pyralis* and multiple copies of the NF-*κ*B consensus sequence fused to a TATA-like promoter region from the herpes simplex virus thymidine kinase promoter, was from Clontech (Mountain View, Calif, USA).

### 2.2. Monolayer Cell Culture and Drug Effect Assays

The biphasic synovial sarcoma cell line SYO-1 and the monophasic human cell line Fuji were kindly provided by Akira Kawai (National Cancer Centre Hospital, Tokyo, Japan), and Kazuo Nagashima (Hokkaido University School of Medicine, Sapporo, Japan), respectively [37, 38]. MTT proliferation and annexin V-FITC/propidium iodide flow cytometry assays were performed as previously described [6].

### 2.3. Protein Quantification

Sample protein concentrations were determined by bicinchoninic acid assay (BCA Protein Assay kit, Pierce, Rockford, Ill, USA) as per manufacturer's instructions. Samples were measured for absorbance at 562 nm in a PowerWaveX enzyme-linked immunoabsorbent assay plate reader from Bio-Tek Instruments (Winooski, Vt, USA).

### 2.4. Lysate Preparation

Total cellular extracts were prepared in lysis buffer (10 mM Tris pH 7.5, 1 mM EGTA, 150 mM NaCl, 1% Triton X-100, 0.5% Nonidet P-40, 1 mM Na_3_VO_4_, and 1 mM PMSF) from 4 × 10^5^ cells, incubated on ice for 20 minutes, and centrifuged at 10 000 g to remove cellular debris. Purified nuclear and cytoplasmic extracts were prepared by using the NE-PER Nuclear and Cytoplasmic Extraction Reagents kit (Pierce, Rockford, Ill, USA).

### 2.5. Immunoblot Analysis

Mouse *α*-acetyl lysine and rabbit *α*-p65 were purchased from Abcam (Cambridge, Mass, USA), mouse *α*-I*κ*B*α* from Cell Signaling (Beverly, Mass, USA), and rabbit *α*-p85 from Upstate Millipore (Charlottesville, Va, USA). Goat *α*-rabbit HRP and goat *α*-mouse HRP secondary antibodies were purchased from Pierce Biotechnology (Rockford, Ill, USA). Protein samples were loaded onto a 10% SDS polyacrylamide gel. Western blotting of the samples was done according to standard procedures. Membranes were incubated in 1 mL of SuperSignal West Femto Luminol/Enhancer Solution with 1 mL Stable Peroxide Buffer from Pierce (Rockford, Ill, USA) at room temperature for 2 minutes and exposed onto photographic film.

### 2.6. Luciferase Assays

SYO-1 cells were plated onto 24-well plates at 4 × 10^4^ cells/well. SYO-1 cells were transfected with 0.3 *μ*g of plasmid/well using FuGENE 6 Transfection reagent (Roche Applied Science, Indianapolis, Ind, USA) as per manufacturer's instructions, and they were treated the following day. After 24 hours of treatment, cells were washed and 100 *μ*L of ice cold 1× Passive Lysis Buffer from the Dual-Luciferase Reporter Assay System kit (Promega, Madison, Wis, USA) was added. Cells were incubated shaking for 20 minutes at room temperature. Samples were aliquoted 3 times at 20 *μ*L/well to a luciferase plate and twice at 10 *μ*L/well to a 96-well plate for protein quantification. Samples were injected with 50 *μ*L/well of LARII from the kit and read on an EG&G Berthold microplate luminometer 96 V (Germany). Samples were normalized to total protein concentration, and average values were compared to vehicle control set to 1.

### 2.7. Synergism Analysis

Synergy was quantified according to the protocol published by Chou and Talalay [39]. The log of the fraction affected/fraction unaffected was plotted as a function of the log of the dose to determine the IC_50_ from the equation log(fa/fu) = *m* log(*D*) − *m* log (IC_50_). Dose responses of drugs in combination were tested at a fixed ratio. Combination index (*CI*) values were obtained from the equation *CIx* = *Dc1x/D1x* + *Dc2x/D2x* + *Dc1xDc2x/D1xD2x*, where *Dc1x* is dose of drug 1 in combination required for achieving x percent of cell inviability. Combination index values below 1 are indication of synergism, near 1 of additivity, and greater than 1 of antagonism. MTT and NF-*κ*B luciferase reporter experiments using the drugs as single agents and in combination were done in triplicates, and all experiments to determine synergism were repeated at least once. Annexin V-FITC apoptosis assays and western blotting assays were likewise repeated once. Statistical analyses on replicates were performed by calculating 95% confidence intervals.

## 3. Results

### 3.1. 17-AAG Synergizes with MS-275 against Synovial Sarcoma in vitro

**Figure 1:**
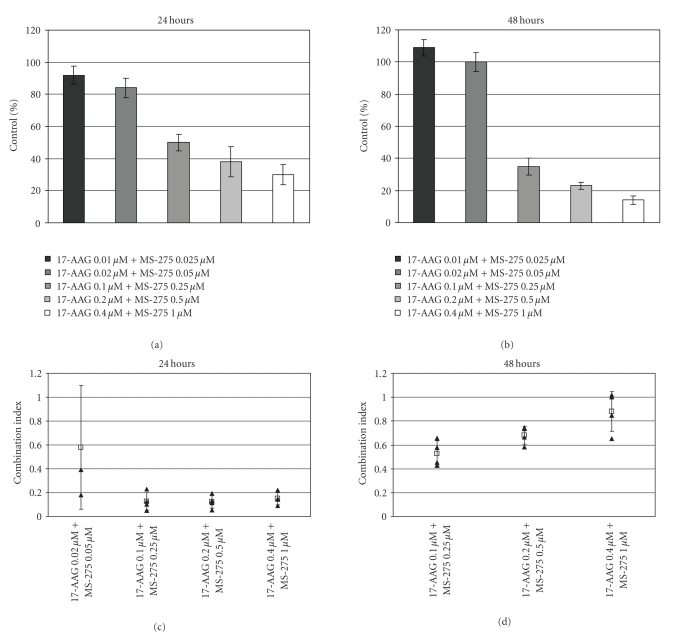
*Effect of 17-AAG and MS-275 in combination on SYO-1 synovial sarcoma monolayer cultures*. Cell survival at (a) 24 hours and (b) 48 hours for drugs combined at a set ratio of 2 parts 17-AAG to 5 parts MS-275. MTT cell proliferation assays were performed for the indicated doses, and survival is plotted relative to vehicle control. Corresponding combination indices are shown for (c) 24 and (d) 48 hours, where values below 1 indicate synergistic drug interactions. Error bars represent 95% confidence intervals. Combination indices were calculated using the Chou and Talalay Median Dose Method (not applicable at low doses where effective cell killing relative to control cell death was not observed).

To determine if the combination of Hsp90 inhibitors and HDAC inhibitors are able to synergize on synovial sarcoma, an in vitro MTT cell proliferation assay was performed on the synovial sarcoma cell lines SYO-1 and Fuji using the Hsp90 inhibitor 17-AAG and the HDAC inhibitor MS-275. Cells were grown in monolayer culture and exposed to varying concentrations of each agent alone to determine IC_50_ values. Both agents are effective at reducing cell proliferation on both synovial sarcoma cell lines in a dose- and time-dependent manner, with IC_50_ values shown in Table 1 for SYO-1. Both are more effective at inhibiting the growth of the synovial sarcoma cell line SYO-1 than equimolar doxorubicin. Whereas 1 *μ*M of doxorubicin reduces the number of viable SYO-1 cells by 7%, 1 *μ*M of 17-AAG and 1 *μ*M of MS-275 reduce the number of cells by 42% and 11%, respectively. Based on these results, the cell lines were then treated in combinations at multiples of a set ratio of 2 parts of 17-AAG to 5 parts of MS-275 and tested for cell proliferation. 17-AAG and MS-275 in combination showed much greater effectiveness at lower doses for reducing cell proliferation on both synovial sarcoma cell lines, in a dose- and time-dependent manner (Figure 1), than either agent alone, with combination index values below 1, indicating synergy.

To confirm synergy, the Annexin V-FITC apoptosis assay was also used at 24 hours and 48 hours. Efficacy of 17-AAG and MS-275 as single agents on synovial sarcoma was confirmed by these assays at all time points in a time- and dose-dependent manner (data not shown). In combination at a set ratio of 2 parts of 17-AAG to 5 parts of MS-275, greater apoptosis was observed at lower doses, again indicating drug synergy. Combination index values were as low as 0.11 and 0.07 for 24 hours and 48 hours, respectively.

### 3.2. Mechanism of Synergy Involves 17-AAG Abrogation of MS-275-Induced NF-*κ*B Activation

**Figure 2:**
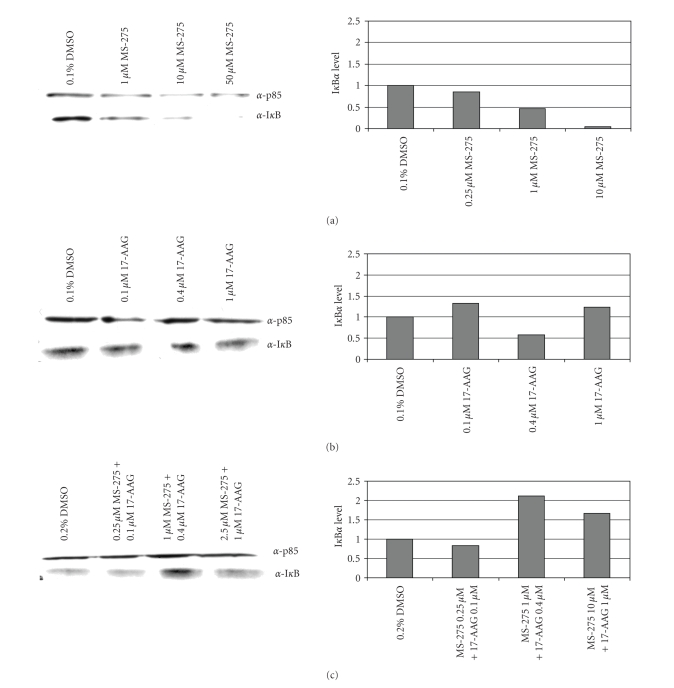
*Dose Response of* I*κ*B*α*. Immuunoblots and quantitation of I*κ*B*α* levels after treatment with (a) MS-275, (b) 17-AAG and (c) Combination. Following 24 hour treatment total lysates were prepared and quantified. 10 *μ*g of lysate was run on an SDS-page gel. By immunoblotting, I*κ*B*α* and p85 (as a loading control) were detected using *α*-I*κ*B*α* and *α*-p85 antibodies. Protein levels following treatment are compared to vehicle control which is set at 1.00.

The I*κ*B*α* complex acts as an NF-*κ*B inhibitor by binding to NF-*κ*B dimers, shuttling them to the cytoplasm, and retaining them there. I*κ*B*α* levels are inversely proportional to NF-*κ*B activation. As an inhibitor of the NF-*κ*B pathway, it has been found that 17-AAG is capable of maintaining levels of I*κ*B*α* [40]. To explore the possibility of synergy through a mechanism involving NF-*κ*B, protein levels of I*κ*B*α* were measured in response to 17-AAG and MS-275 individually and in combination with synovial sarcoma cells. By western blot, I*κ*B*α* levels decrease with MS-275 treatment in a dose-dependent manner, indicating activation of the NF-*κ*B pathway; an effect which is abrogated by adding 17-AAG (Figure 2).

**Figure 3:**
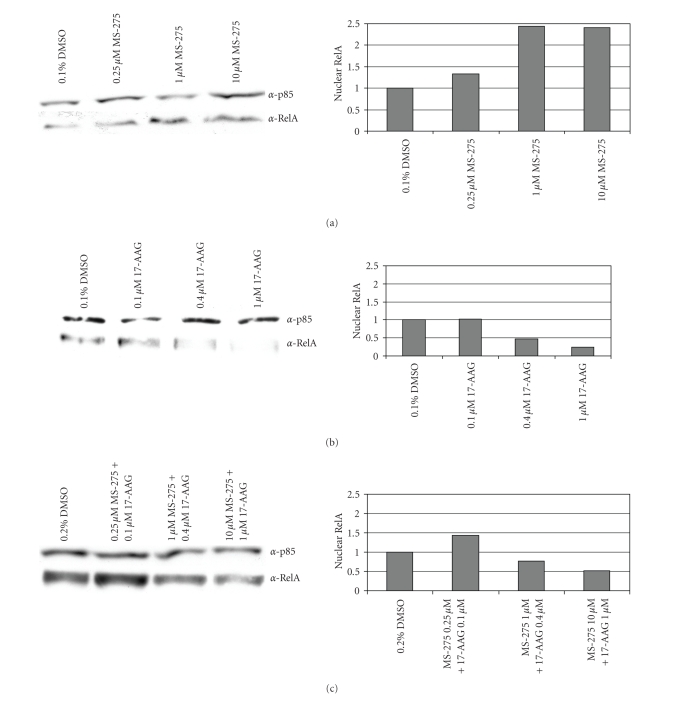
*Dose Response of RelA*. Immunoblots and quantitation of nuclear RelA after treatment with (a) MS-275, (b ) 17-AAG and (c) Combination. Following 24 hour treatment, nuclear and cytoplasmic extracts were prepared and quantified. 15 *μ*g were run on an SDS-page gel. Using immunoblotting techiniques, RelA and p85 (as a loading control) were detected using *α*-RelA and *α*-p85 antibodies. Protein levels following treatment are compared to vehicle control which is set at 1.00.

NF-*κ*B activation requires relocation of NF-*κ*B heterodimers from the cytoplasm to the nucleus before it can mediate its transcriptional effects on cell survival. In synovial sarcoma cells, we find that nuclear levels of the RelA subunit of NF-*κ*B increase during MS-275 treatment, but decrease in the presence of 17-AAG as a single agent or in combination with MS-275 (Figure 3).

**Figure 4:**
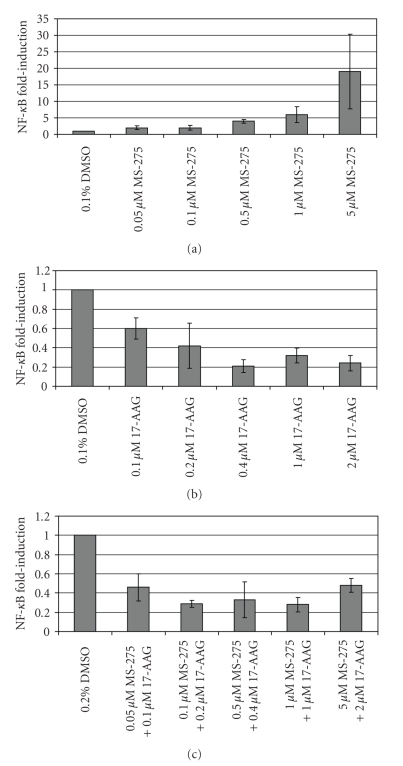
*Transcriptional activation of NF-*κ*B luciferase reporter*. NF-*κ*B induction following treatment with (a) MS-275, (b) 17-AAG, and (c) Combination. SYO-1 cells were grown as monolayer cultures in 24 well plates and transfected with 0.3 *μ*g of NF-*κ*B luciferase reporter plasmid. Cells were treated the following day for 24 hours and lysed with passive lysis buffer. Samples were aliquoted to plates and simultaneously assayed for luminosity by injection with 50 *μ*l/well of LARII reagent and protein quantity by copper sulfate/bichionic acid assay. Readings for luminosity were normalized to protein concentration and vehicle control was set to 1.00. Error bars represent 95% confidence intervals for three replicate measurements.

Finally, we looked at the transcriptional potential of NF-*κ*B following treatment with these drugs using an NF-*κ*B luciferase reporter (Figure 4). MS-275 dramatically increases NF-*κ*B transcriptional activity (almost 20-fold at higher doses), whereas 17-AAG as a single agent decreases transcriptional activity. In the combination, the 17-AAG effect predominates, and there is a net inhibition of NF-*κ*B transcriptional activity. Similar results were found with Fuji synovial sarcoma cells (data not shown).

### 3.3. Histone Deacetylase 3 (HDAC3) siRNA Knockdown Has Similar Effects to MS-275

**Figure 5:**
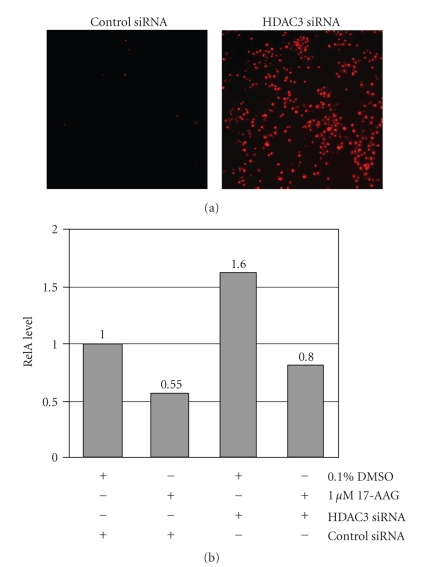
*HDAC3 siRNA knockdown recapitulates the effects HDAC inhibitors on synovial sarcoma cells*. (a) 50 nM HDAC3 siRNA promotes extensive cell death at 24 hours, as shown by staining of a large inviable cell fraction by 500 ng/mL propidium iodide, where virtually all cells exposed to equimolar scrambled siRNA control are viable. (b) Densitometric quantitation of Western blots for nuclear RelA, expressed relative to control siRNA transfected cells treated with vehicle (1.0).

The RelA subunit of NF-*κ*B is acetylated by HDAC3 [22], reducing its ability to interact with its inhibitor I*κ*B*α*. All HDAC inhibitors previously shown to kill synovial sarcoma cells [16–18] include HDAC3 among their specific targets. Therefore, we hypothesized that our observations on MS-275 (killing synovial sarcoma cells, but activating NF-*κ*B in a manner which can be blocked with 17-AAG) might be explained specifically by HDAC3 inhibition. We find that siRNA directed against HDAC3 kills SYO-1 cells, whereas the scrambled siRNA control does not (Figure 5(a)). Furthermore, HDAC3 knockdown induces RelA nuclear translocation, and this effect is largely abrogated by adding 17-AAG (Figure 5(b)). Thus, the same effects on cell survival and NF-*κ*B activation are seen with HDAC3 siRNA knockdown as with the HDAC inhibitor drug MS-275.

### 3.4. MS-275 Synergizes with NF-*κ*B Inhibitors

The compound BAY 11-7085 is a commercially available inhibitor of NF-*κ*B [41]. In our NF-*κ*B luciferase reporter assay system, BAY 11-7085 strongly represses NF-*κ*B activity in SYO-1 cells with an IC_50_ of 4.9 *μ*M at 24 hours. The combination of BAY 11-7085 and MS-275 showed combination index values as low as 0.26 for 24 hours and 0.25 for 48 hours, indicating synergism between this NF-*κ*B inhibitor and the HDAC inhibitor MS-275 (Table 2).

## 4. Discussion

In current clinical practice, available systemic therapies for synovial sarcoma have limited effectiveness and have not been definitively proven to increase cure rates. Recent research building on gene expression profiling data has identified several promising agents active against synovial sarcoma [42]. 17-AAG is effective against synovial sarcoma preclinical models, but clinical trials in other tumors have shown some toxicity at higher doses including evidence of liver toxicity, optic neuritis, dyspnea, fatigue, nausea, vomiting, anorexia, diarrhea, anemia, and low grade fever [43, 44]. Similarly, in high doses, MS-275 has shown such toxicity as nausea, vomiting, anorexia, fatigue, hypoalbuminemia, and hypocalcemia in clinical trials [45].The HDAC inhibitor romidepsin has additionally been associated with atrial fibrillation, tachycardia, and in one case a cardiac sudden death [46]. Synergism would allow lower doses to be used. While synergism has been demonstrated by others between 17-AAG and HDAC inhibitors in myelogenous leukemia cells [10, 28], our study is the first to demonstrate that the orally available agent MS-275 synergizes with 17-AAG. This is also the first demonstration of synergism between Hsp90 and HDAC inhibitors in synovial sarcoma; an often fatal disease wherein each of these drug classes has recently been demonstrated to show activity in vitro [6, 16]. Here we have shown that combinations of the Hsp90 inhibitor 17-AAG and the HDAC inhibitor MS-275 are synergistic, requiring as little as ninefold less drug to achieve equivalent effects in vitro against synovial sarcoma models.

Our results provide evidence that synergism between 17-AAG and MS-275 might be mediated by the prosurvival NF-*κ*B pathway. Evidence from microarray expression profiling has demonstrated that the NF-*κ*B activator RIPK4 is highly upregulated in synovial sarcoma, and the data presented here shows that the RelA subunit of NF-*κ*B is found in the nucleus of untreated synovial sarcoma cells, suggesting baseline activation of NF-*κ*B in this malignancy. In addition, the TLE transcriptional corepressor, which is expressed at extremely high levels in synovial sarcoma cells [47], forms a complex with HDACs at NF-*κ*B target sites [48]. Consistent with a role for NF-*κ*B in synovial sarcoma growth, we show that a chemical inhibitor of NF-*κ*B (BAY 11-7085) has in vitro activity against synovial sarcoma cells as a single agent. Although additional mechanisms may contribute to the observed synergy, in separate experiments, we found that neither 17-AAG nor HDAC inhibitors altered the level of SYT-SSX protein expression, nor are Hsp90 levels or acetylation status altered by HDAC inhibitor treatment (data not shown).

In conclusion, the Hsp90 inhibitor 17-AAG and the HDAC inhibitor MS-275 have synergistic, antiproliferative, and proapoptotic effects on synovial sarcoma in vitro. 17-AAG and MS-275 in combination reverse the activation of NF-*κ*B seen with MS-275 alone, as measured by levels of the NF-*κ*B inhibitory I*κ*B*α* complex. Net effects of these drugs on nuclear levels of the active NF-*κ*B subunit RelA and on NF-*κ*B luciferase reporter transcription are consistent with these findings. The observed effects of MS-275 on NF-*κ*B can be recapitulated by knocking down HDAC3; an enzyme which includes RelA as one of its nonhistone substrates and which is a target of MS-275, depsipeptide, and several other HDAC inhibitors. In addition, the NF-*κ*B inhibitor BAY 11-7085 is also synergistic with MS-275. Agents inhibiting NF-*κ*B in combination with the HDAC inhibitor MS-275 show promising in vitro activity against synovial sarcoma; an often-fatal disease of young adults for which development of truly effective systemic therapies is needed.

## Figures and Tables

**Table 1:** Comparison of IC_50_ values at 24- and 48 hour Timepoints for MS-275 and 17-AAG as single agents and in combination using MTT assay.

Timepoint (hrs)	IC_50_ average			
	MS-275	17-AAG	Combination (*μ*M)	
	(*μ*M)	(*μ*M)	MS-275	17-AAG
24	4.5	2.1	0.6	0.25
48	0.56	0.32	0.3	0.12

**Table 2:** Combination of HDAC and NF-*κ*B inhibitors against synovial sarcoma cells.

Timepoint (hrs)	IC_50_ average			
	MS-275	BAY 11-7085	Combination (*μ*M)	
	(*μ*M)	(*μ*M)	MS-275	BAY 11
24	4.5	3.5	0.45	1.4
48	0.56	3.4	0.27	0.8
